# Research hotspots and development trends in molecular imaging of glioma (2014–2024): A bibliometric review

**DOI:** 10.1097/MD.0000000000042862

**Published:** 2025-06-20

**Authors:** Hui Zhou, Yilin Luo, Shiguang Li, Guoping Zhang, Xianchun Zeng

**Affiliations:** a Department of Radiology, The Affiliated Jinyang Hospital of Guizhou Medical University, Guiyang, China; b Department of Nuclear Medicine, Guizhou Provincial People’s Hospital, Guiyang, China.

**Keywords:** bibliometric analysis, citespace, glioma, molecular imaging

## Abstract

**Background:**

This study aims to explore research hotspots and development trends in molecular imaging of glioma from 2014 to 2024.

**Methods:**

A total of 2957 publications indexed in the web of science core collection (WoSCC) were analyzed using bibliometric techniques. To visualize the research landscape, co-citation clustering, keyword analysis, and technological trend mapping were performed using CiteSpace and Excel.

**Results:**

Publication output peaked in 2021. Emerging research trends included the integration of radiomics and artificial intelligence and the application of novel imaging modalities such as positron emission tomography and magnetic resonance spectroscopy. Significant progress was observed in blood–brain barrier disruption techniques and the development of molecular probes, especially those targeting IDH and MGMT mutations.

**Conclusion:**

Molecular imaging has been pivotal in advancing glioma research, contributing to improved diagnostic accuracy and personalized treatment strategies. However, challenges such as clinical translation and standardization remain. Future studies should focus on integrating advanced technologies into routine clinical practice to enhance patient care.

## 1. Introduction

Gliomas are the most common and aggressive malignant tumors in the central nervous system.^[1]^ Their high incidence and mortality have made them a key focus of neuro-oncological research. However, gliomas’ complex molecular mechanisms and marked heterogeneity present significant challenges for accurate diagnosis, effective treatment, and prognostic evaluation.^[2–4]^ Molecular imaging, which enables the visualization of biological processes at the molecular and cellular levels,^[5]^ is pivotal in the precise diagnosis and individualized treatment of gliomas. It also enhances understanding of the biological and pathological mechanisms underlying these tumors.^[6,7]^

In recent years, bibliometric analysis has emerged as a valuable approach for mapping research landscapes,^[8]^ providing insights into hotspots and advancements in fields such as immunotherapy, nanotechnology, and radiomics.^[9–12]^ However, existing bibliometric studies often focus on specific technologies or therapeutic approaches, devoting limited attention to glioma molecular imaging – a rapidly growing interdisciplinary field. This gap underscores the need for a systematic bibliometric analysis to elucidate current research trends and priorities in glioma molecular imaging.

Accordingly, this study aims to summarize progress in glioma molecular imaging, identify future directions, and offer strategic guidance for advancing molecular imaging technologies in glioma research.

## 2. Methodology

### 2.1. Study design

This bibliometric analysis aims to evaluate the status, research hotspots, and development trends of molecular imaging technology for gliomas from 2014 to 2024.

### 2.2. Data acquisition and search strategy

Citation data were retrieved from the web of science core collection (WoSCC), specifically focusing on the Science Citation Index Expanded from 2014 to 2024.^[13,14]^ The search included sub-databases such as the science citation index expanded (coverage: 2014 to present), current chemical reactions (coverage: 1985 to present), and Index Chemicus (coverage: 1993 to present).

Thematic keywords were selected based on relevance to glioma and molecular imaging (7). The search query was: TS = (“Glioma” OR “Glial Tumor” OR “Astrocytoma” OR “Glioblastoma” OR “Diffuse Glioma” OR “Oligodendroglioma” OR “Ependymoma”) AND (“Molecular Imaging” OR “positron emission tomography (PET)” OR “Single Photon Emission Computed Tomography” OR “Magnetic resonance spectroscopy (MRS)” OR “chemical exchange saturation transfer (CEST)” OR “Optical Imaging” OR “Fluorescence Imaging” OR “Bioluminescence Imaging” OR “Near-Infrared Imaging” OR “Photoacoustic Imaging” OR “Radionuclide Imaging” OR “Radiotracer Imaging” OR “Hyperpolarized Magnetic Resonance Imaging” OR “Ultrasound with Microbubble Imaging”).

**Figure 1.**
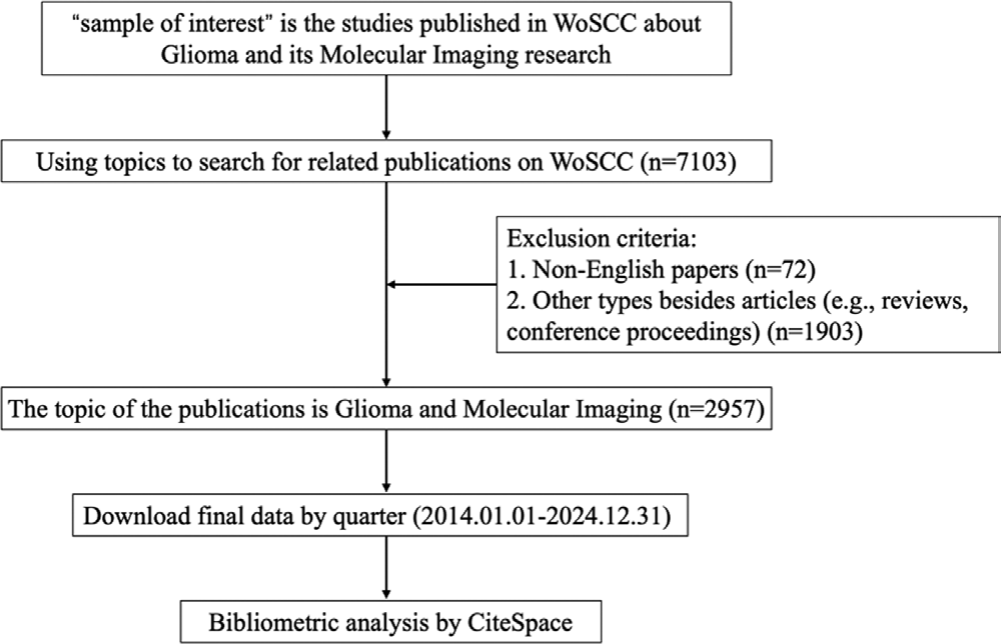
Flowchart of the literature selection process for bibliometric analysis. A total of 7103 records related to glioma and molecular imaging were retrieved from the WoSCC database. After excluding non-English articles (n = 72) and non-original research types such as reviews and conference papers (n = 1903), 2957 articles were included for further bibliometric analysis using CiteSpace, covering the period from January 1, 2014, to December 31, 2024.

The search was limited to titles, abstracts, and keywords (TS field) and included only English-language articles classified as “Article.” The initial search yielded 7103 articles; filtering for article type reduced the results to 5043, followed by a limitation to English-language publications (4971 articles). Finally, applying the period of 2014 to 2024 resulted in 2957 articles. These articles were downloaded as plain text files with full records and citations and subsequently analyzed using CiteSpace V 6.4.R1 (Drexel University, Philadelphia). Journal impact factors (JIF) and subject categories were obtained from the Journal Citation Report 2023 (Clarivate, https://www.webofscience.com/). Figure 1 provides a flowchart of the search strategy and selection procedures.

### 2.3. Bibliometric analysis

Bibliometric analysis and visualization were conducted using CiteSpace (version 6.4.R1), the online bibliometric analysis platform (https://bibliometric.com/app), and Microsoft Excel (version 16.92) for data management. CiteSpace facilitates the exploration of knowledge domains and their evolutionary dynamics through various visualization techniques.^[15,16]^ It identifies research frontiers, detects critical turning points, and visualizes collaboration networks and thematic trends. Betweenness centrality was used to gauge the importance of nodes within networks; nodes with scores ≥ 0.1 were marked by purple rings.^[17]^

Data were exported from WoS in plain text format, including full records and cited references (up to 500 records per batch), and then imported into CiteSpace for analysis. The key analyses included literature mapping, coauthorship, keyword co-occurrence, and journal publication analysis. JIF from the Journal Citation Reports were integrated to evaluate the quality and influence of publications. Additionally, the H-index available in WoS was employed to measure the productivity and citation impact of authors, journals, and institutions.

To identify key research trends and influential elements, this study adopted the TOP N% algorithm with N% = 10%,^[18,19]^ effectively minimizing bias from annual publication fluctuations and enabling a more accurate depiction of temporal research priorities.

In addition to CiteSpace, the online bibliometric platform (https://bibliometric.com/app) was used to examine national and regional collaborations, and Excel was used for data organization. This study provides a comprehensive bibliometric analysis of research developments in glioma molecular imaging by integrating multiple tools and metrics such as JIF, H-index, and the TOP N% algorithm.

## 3. Results

### 3.1. Date of publication analysis

A total of 2957 research articles on glioma and molecular imaging were published between 2014 and 2024, yielding 69,876 citations, with an average citation of 23.62 per article. The H-index reached 99, indicating that at least 99 articles were cited 99 times or more.

**Figure 2.**
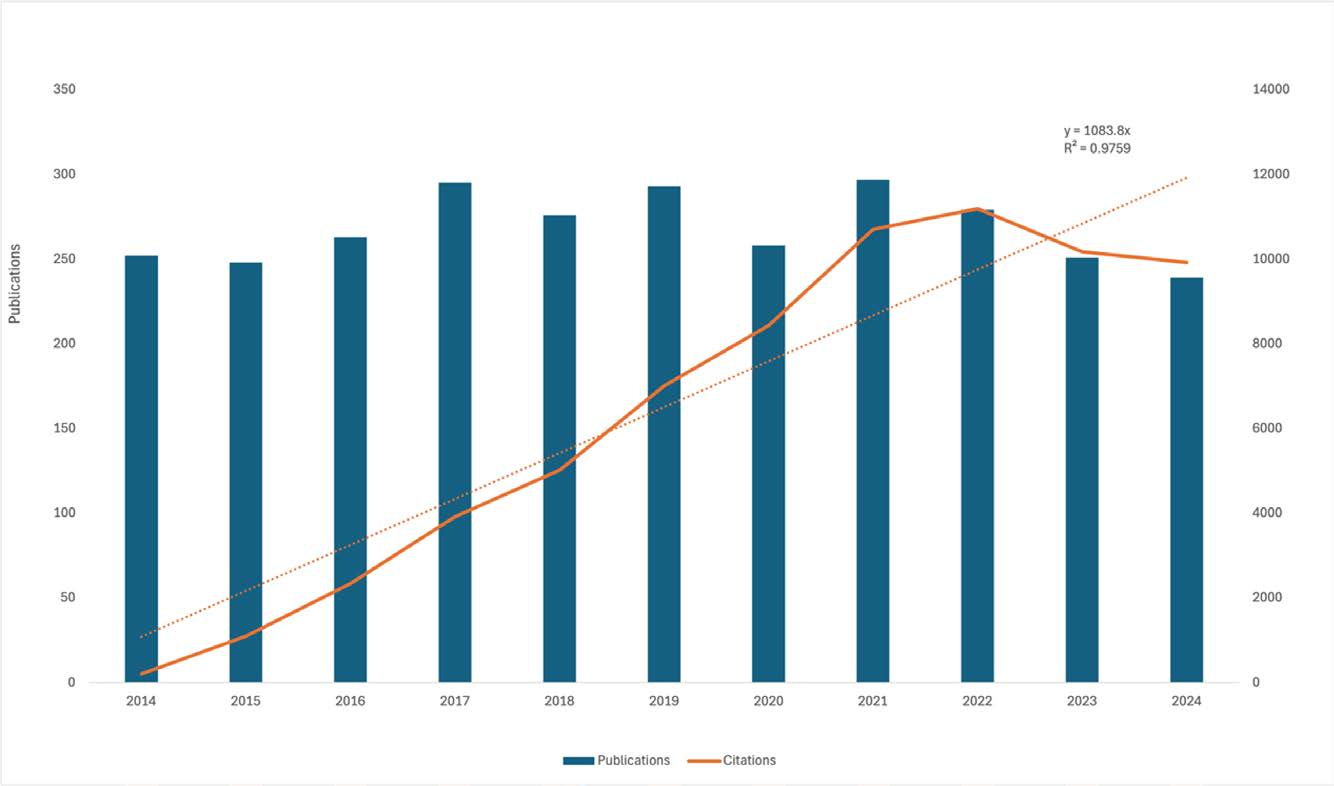
Annual number of publications and total citations related to glioma molecular imaging from 2014 to 2024. The bar chart shows the yearly publication count, while the line represents the cumulative citations. A linear trend line is also plotted to illustrate the overall citation growth (*R*^2^ = 0.9759).

As shown in Figure 2, between 2014 and 2016, the annual publication count remained steady, ranging from 248 to 263 articles. Beginning in 2017, the publication volume increased markedly, peaking at 297 articles in 2021. A slight decline was observed thereafter, with 239 articles recorded in 2024.

Citation trends mirrored publication growth, rising from 205 in 2014 to 11,188 in 2022, which was the highest annual citation count within the study period. Although the citation numbers dipped slightly in 2023 and 2024, they remained relatively high, at 10,174 and 9923 citations, respectively.

### 3.2. Analysis of country, institutional distribution, and funding agencies

Between 2014 and 2024, the United States published the most articles on glioma molecular imaging, with 983 publications (33.24%), an H-index of 82, and a centrality of 0.35. China followed with 659 publications (22.29%), an H-index of 62, and a centrality of 0.19. Germany ranked third with 426 publications (14.41%), an H-index of 65, and an average citation of 32.03 per article. Japan (242 publications, 8.18%), France (174 publications, 5.88%), and the Netherlands (93 publications, 3.15%) also made notable contributions; the Netherlands attained the highest average citation count of 35 per article (Table 1).

**Table 1:** Top 10 countries/regions contributing to glioma molecular imaging research from 2014 to 2024, ranked by number of publications.

Rank	Country/region	Counts	% of 2957	Citations	Average citations	H-index	Centrality
1	USA	983	33.243	30,660	31.19	82	0.35
2	China	659	22.286	16,199	24.58	62	0.19
3	Germany	426	14.406	13,644	32.03	65	0.16
4	Japan	242	8.184	4536	18.74	36	0.01
5	France	174	5.884	3360	19.31	33	0.09
6	Italy	146	4.937	3528	24.16	29	0.2
7	England	138	4.667	3361	24.46	30	0.17
8	Canada	100	3.382	2953	29.53	27	0.04
9	South Korea	97	3.28	2277	23.47	27	0.02
10	Netherlands	93	3.145	3255	35	30	0.12

Metrics include total citation count, average citations per article, H-index, and betweenness centrality.

Funding agency analysis showed that the United States Department of Health and Human Services (547 articles, 35.87 average citations, H-index 70) and the National Institutes of Health (546 articles, 35.92 average citations, H-index 70) were the leading agencies (Table 2). The National Natural Science Foundation of China ranked third (399 articles, 29.64 average citations, H-index 57). Other prominent agencies included the NIH National Cancer Institute (215 articles, 32.65 average citations), the German Research Foundation (96 articles, 21.46 average citations), and the European Union (67 articles, 17.63 average citations).

**Table 2:** Top 10 funding agencies supporting glioma molecular imaging research from 2014 to 2024.

Funding agencies	Articles	Citations	Average citations	H-Index
United States Department of Health and Human Services	547	19,622	35.87	70
NIH USA	546	19,614	35.92	70
NSFC	399	11,945	29.64	57
NIH NCI	215	7019	32.65	41
MEXT	132	2323	17.6	23
Japan Society for the Promotion of Science	121	2126	17.57	23
Grants in Aid for Scientific Research KAKENHI	117	2111	18.04	23
DFG	96	2060	21.46	25
EU	67	1181	17.63	19
National Key Research Development Program of China	49	1439	29.37	23

Metrics include the number of funded articles, total citations, average citations per article, and H-index.

DFG = German Research Foundation, EU = European Union, MEXT = Ministry of Education Culture Sports Science and Technology, NCI = National Cancer Institute, NIH = National Institutes of Health, NSFC = National Natural Science Foundation of China.

Institutional analysis revealed that the Helmholtz Association contributed the largest number of publications (229, 7.74% of the total), with an average citation of 33.13 and an H-index of 49 (Table 3). The University of California System (149 publications, average citation 34.38) exhibited the highest centrality of 0.57, indicating its key role in global collaborations. The German Cancer Research Center (DKFZ) followed with 129 publications (4.36%) and an H-index of 34. Other leading institutions included Harvard University (101 publications, 41.66 average citations, H-index 38) and the Chinese Academy of Sciences (96 publications, 46.31 average citations, H-index 35).

**Table 3:** Top 10 research institutions publishing glioma molecular imaging studies from 2014 to 2024.

Rank	Institution	Counts	% of 2957	Citations	Average citations	H-index	Centrality
1	Helmholtz Association	229	7.744	7586	33.13	49	0.46
2	University of California System	149	5.039	5122	34.38	39	0.57
3	German Cancer Research Center DKFZ	129	4.363	3695	28.64	34	0.22
4	French National Institute of Health and Medical Research	119	4.024	1988	16.71	27	0.05
5	Centre National de la Recherche Scientifique CNRS	113	3.821	2234	19.77	28	0.19
6	University of Munich	105	3.551	3805	36.24	32	0.07
7	Harvard University	101	3.416	4208	41.66	38	0.35
8	Chinese Academy of Sciences	96	3.247	4446	46.31	35	0.14
9	University of Cologne	84	2.841	3666	43.64	32	0.02
10	University of Texas System	83	2.807	3335	40.18	30	0.02

Metrics include publication count, total citations, average citations per article, H-index, and centrality score.

### 3.3. Analysis of author and co-cited author distribution

An examination of author contributions showed that Galldiks, Norbert, ranked first with 72 publications, 3426 citations, and an average of 47.58 citations per article, yielding an H-index of 30 (Table 4). Langen, Karl-Josef, followed with 68 publications and 2774 citations (H-index 29). Other notable authors included Stoffels, Gabriele (45 publications, 1698 citations), Lohmann, Philipp (45 publications, 1334 citations), and Albert, Nathalie L. (44 publications, 1319 citations).

**Table 4:** Top 10 most productive authors in glioma molecular imaging research from 2014 to 2024, ranked by publication count.

Rank	Author	Counts	Citations	H-index	coauthor	Citations
1	Galldiks	72	3426	30	Louis	693
2	Langen	68	2774	29	Stupp	529
3	Stoffels	45	1698	21	Wen	421
4	Lohmann	45	1334	21	Galldiks	360
5	Albert	44	1319	19	Ostrom	264
6	Bartenstein	44	1187	19	Unknown-	251
7	Shah	43	1569	21	Albert	230
8	Unterrainer	34	810	17	Weller	161
9	Fink	34	1416	19	Law	139
10	Ellingson	32	956	16	Pauleit	129

Citation metrics, H-index, and major coauthors are also presented.

### 3.4. Analysis of cited journals

A total of 575 journals published articles in this field. The top 3 journals by publication volume were European Journal of Nuclear Medicine and Molecular Imaging (1034 articles), Oncology (627 articles), and Clinical Neurology (481 articles). In co-citation analyses, Neuro-Oncology was cited most frequently (1589 citations), followed by the Journal of Neuro-Oncology (1399 citations) and the Journal of Nuclear Medicine (1299 citations) (Table 5).

**Table 5:** Top subject categories and co-cited journals in glioma molecular imaging research from 2014 to 2024.

Rank	Count	% of 2957	Journal	JIF	JIF (quartile) 2023	Co-cited journal	Citation	Centrality	JIF	JIF (quarile) 2023
1	1034	34.956	European Journal of Nuclear Medicine and Molecular Imaging	8.6	1	Neuro-Oncology	1589	0.11	16.4	1
2	627	21.197	Oncology	3.2	2	Journal of Neuro-Oncology	1399	0.06	3.2	2
3	481	16.261	Clinical Neurology	9.1	1	Journal of Nuclear Medicine	1299	0.13	9.1	1
4	187	6.322	Chemistry Multidisciplinary	16.4	1	Clinical Cancer Research	1078	0.1	10	1
5	168	5.68	Multidisciplinary Sciences	3.8	1	Cancer Research	1061	0.11	12.5	1
6	164	5.544	Medicine Research Experimental	2.7	2	European Journal of Nuclear Medicine and Molecular Imaging	1021	0.11	8.6	1
7	159	5.375	Surgery	4.5	1	PLOS ONE	1003	0.03	2.9	3
8	157	5.308	Neurosciences	3	2	American Journal of Neuroradiology	910	0.08	3.1	3
9	148	5.003	Nanoscience Nanotechnology	3.5	2	Journal of Clinical Oncology	896	0.06	42.1	1
10	143	4.834	Biochemistry Molecular Biology	2.9	3.3	New England Journal of Medicine	870	0.02	96.2	1
11	141	4.768	Pharmacology Pharmacy	3	2	Journal of Neurosurgery	869	0.05	3.5	2
12	126	4.261	Materials Science Multidisciplinary	10	1	Journal of Neurosurgery	849	0.05	3.5	1
13	86	2.908	Biochemical Research Methods	3.1	1	Proceedings of the National Academy of Sciences of the United States of America (PNAS)	834	0.12	9.4	1
14	86	2.908	Biophysics	1.9	3	Acta Neuropathologica	741	0.04	9.3	1
15	86	2.908	Engineering Biomedical	12.4	1	The Lancet Oncology	693	0.03	58.7	1
16	81	2.739	Cell Biology	10.4	1	Scientific Reports (UK)	677	0.03	3.8	2
17	73	2.469	Neuroimaging	4.7	1	Nature Medicine	655	0.03	58.7	1
18	73	2.469	Spectroscopy	5.168	2	Neurosurgery	653	0.02	3.9	2
19	66	2.232	Chemistry Physical	3.6	1	International Journal of Radiation Oncology, Biology, Physics	601	0.02	2.7	3
20	65	2.198	Chemistry Medicinal	1.3	3	Nature	578	0.02	50.5	1

The table includes journal impact factors (JIF), JIF quartiles, citation counts, and centrality, highlighting both source journals and frequently co-cited journals in the field.

JIF = journal impact factor.

### 3.5. Analysis of references

Among the top 10 most-cited articles (2014–2024), several were published in high-impact journals such as Cell and Advanced Materials and specialized journals like Neuro-Oncology. Details of the top 10 most-cited references are listed in Table 6. The most-cited article, published in Cell, received 630 citations, underscoring the critical role of top-tier journals in propelling research in this domain. Articles published around 2014 and earlier accrued higher citation counts, but more recent publications (2020 onward) showed strong citation growth potential. Key research themes included neuro-oncology, radiomics, and molecular metabolic biomarkers, reflecting the multidisciplinary nature of this field.

**Table 6:** Top 10 most-cited references in glioma molecular imaging literature from 2014 to 2024.

RANK	Reference	Citations	Joumal	JIF	First author	Time
1	Acetate is a bioenergetic substrate for human glioblastoma and brain metastases	630	Cell	45.5	Mashimo	December 18, 2014
2	Response assessment in neuro-oncology working group and European Association for neuro-oncology recommendations for the clinical use of PET imaging in gliomas	529	Neuro-Oncology	16.4	Albert	September 1, 2016
3	Self-targeting fluorescent carbon dots for diagnosis of brain cancer cells	447	ACS Nano	15.8	Zheng	November 1, 2015
4	Through scalp and skull NIR-II photothermal therapy of deep orthotopic brain tumors with precise photoacoustic imaging guidance	370	Advanced Materials	27.4	Guo	August 29, 2018
5	Safety and tumor specificity of cetuximab-IRDye800 for surgical navigation in head and neck cancer	379	Clinical Cancer Research	10	Rosenthal	August 15, 2015
6	Joint EANM/EANO/RANO practice guidelines/SNMMI procedure standards for imaging of gliomas using PET with radiolabelled amino acids and [18F]FDG: version 1.0	352	European Journal of Nuclear Medicine and Molecular Imaging	8.6	Law	March 13, 2019
7	Amide proton transfer imaging of adult diffuse gliomas: correlation with histopathological grades	344	Neuro-Oncology	16.4	Togao	March 1, 2014
8	Targeted tumor theranostics in mice via carbon quantum dots structurally mimicking large amino acids	285	Nature Biomedical Engineering	26.8	Li	March 30, 2020
9	T2-FLAIR mismatch, an imaging biomarker for IDH and 1p/19q status in lower- grade gliomas: a TCGA/TCIA project	285	Clinical Cancer Research	10	Patel	October 15, 2017
10	Dual-targeting upconversion nanoprobes across the blood–brain barrier for magnetic resonance/fluorescence imaging of intracranial glioblastoma	280	ACS Nano	15.8	Ni	February 1, 2014

Listed are article titles, citation counts, journal names, impact factors (JIF), first authors, and publication dates.

JIF = journal impact factor.

**Figure 3.**
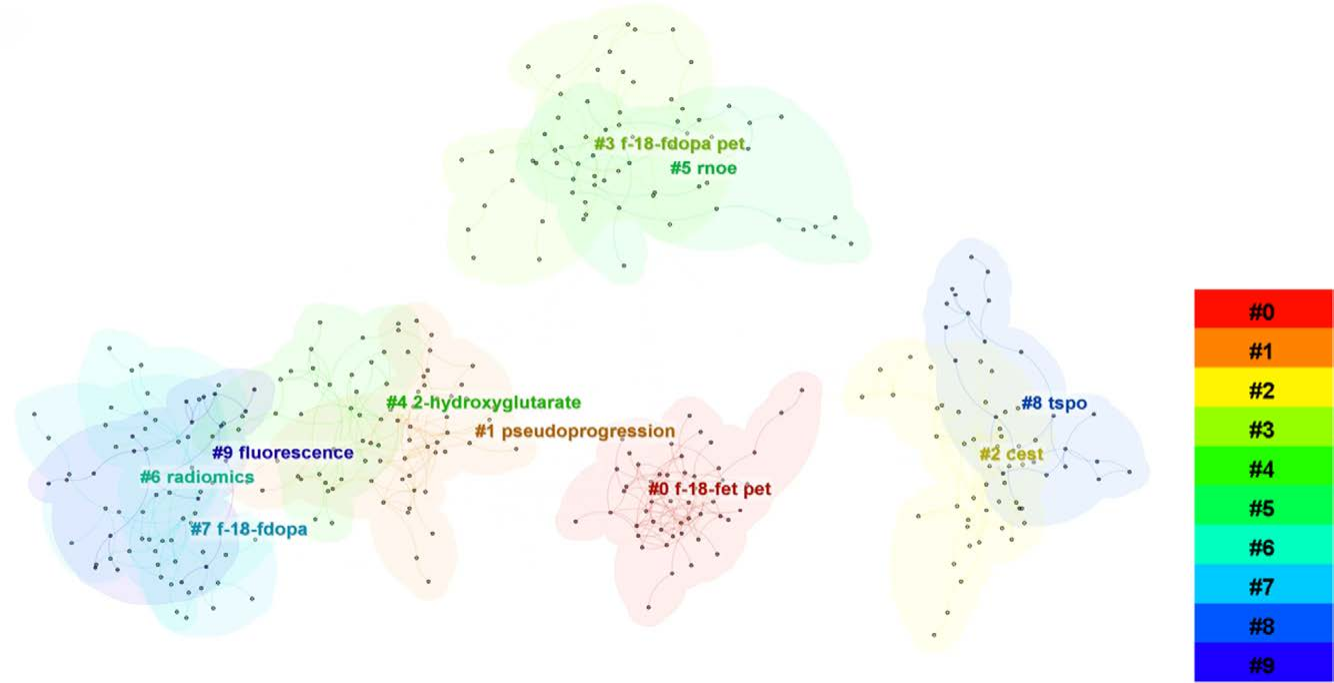
Collaboration network among countries contributing to glioma molecular imaging research. Each node represents a country, with node size indicating the number of publications. The connecting lines indicate international collaborations, and the thickness of the lines reflects the strength of cooperative relationships.

**Figure 4.**
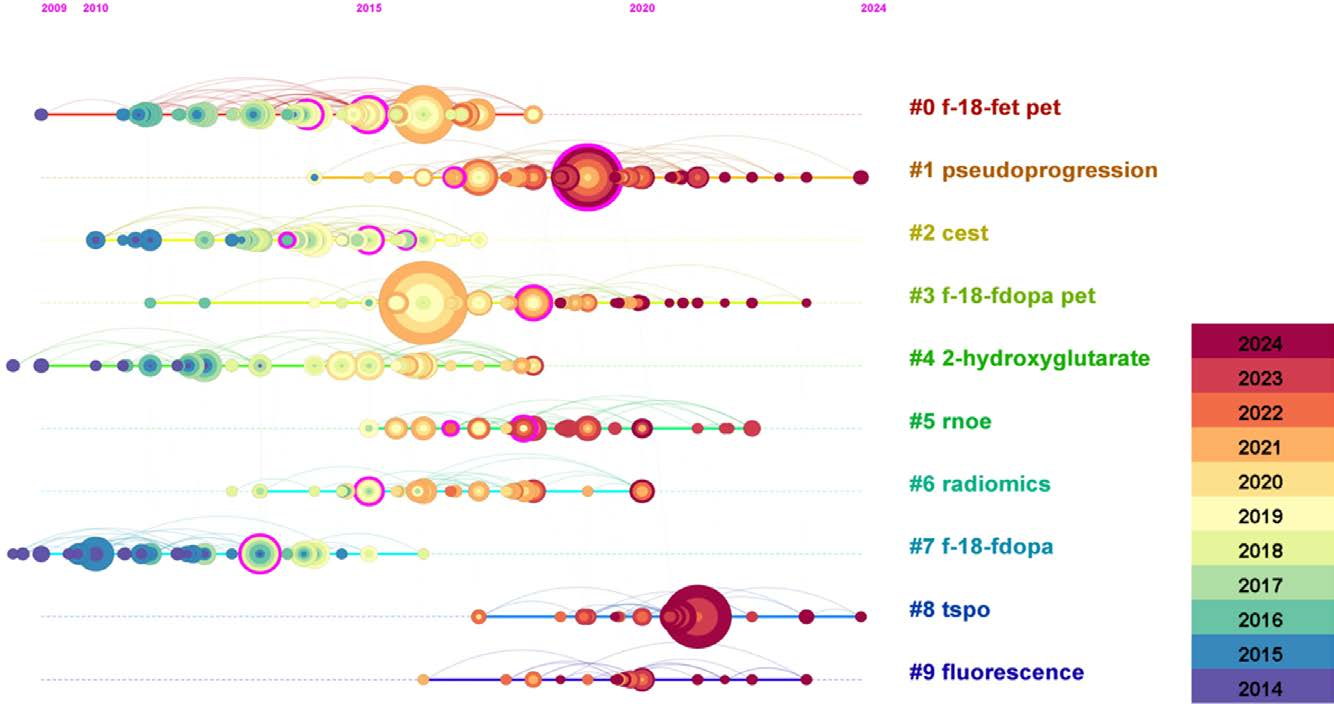
Institutional collaboration network in the field of glioma molecular imaging. Nodes represent research institutions, with node size proportional to the number of publications. Links between nodes indicate inter-institutional collaborations, and thicker lines reflect stronger cooperative intensity.

Co-citation analysis identified 10 major clusters, numbered by importance (Fig. 3). The largest was Cluster #0 (F-18-FET PET), underscoring the prominence of radioactive tracers in molecular imaging. Cluster #1 (pseudoprogression) addressed diagnostic challenges in tumor assessment, while Clusters #6 (radiomics) and #4 (2-hydroxyglutarate) highlighted rapid research growth in radiomics and metabolic biomarker detection. The timeline distribution (Fig. 4) indicated that early research clusters, such as #3 (F-18-FDOPA PET) and #9 (fluorescence), were active between 2010 and 2015, gradually giving way to more recent topics (#0 F-18-FET PET, #1 pseudoprogression, #6 radiomics, and #4 2-hydroxyglutarate).

### 3.6. Analysis of keywords and co-occurrence clusters

**Figure 5.**
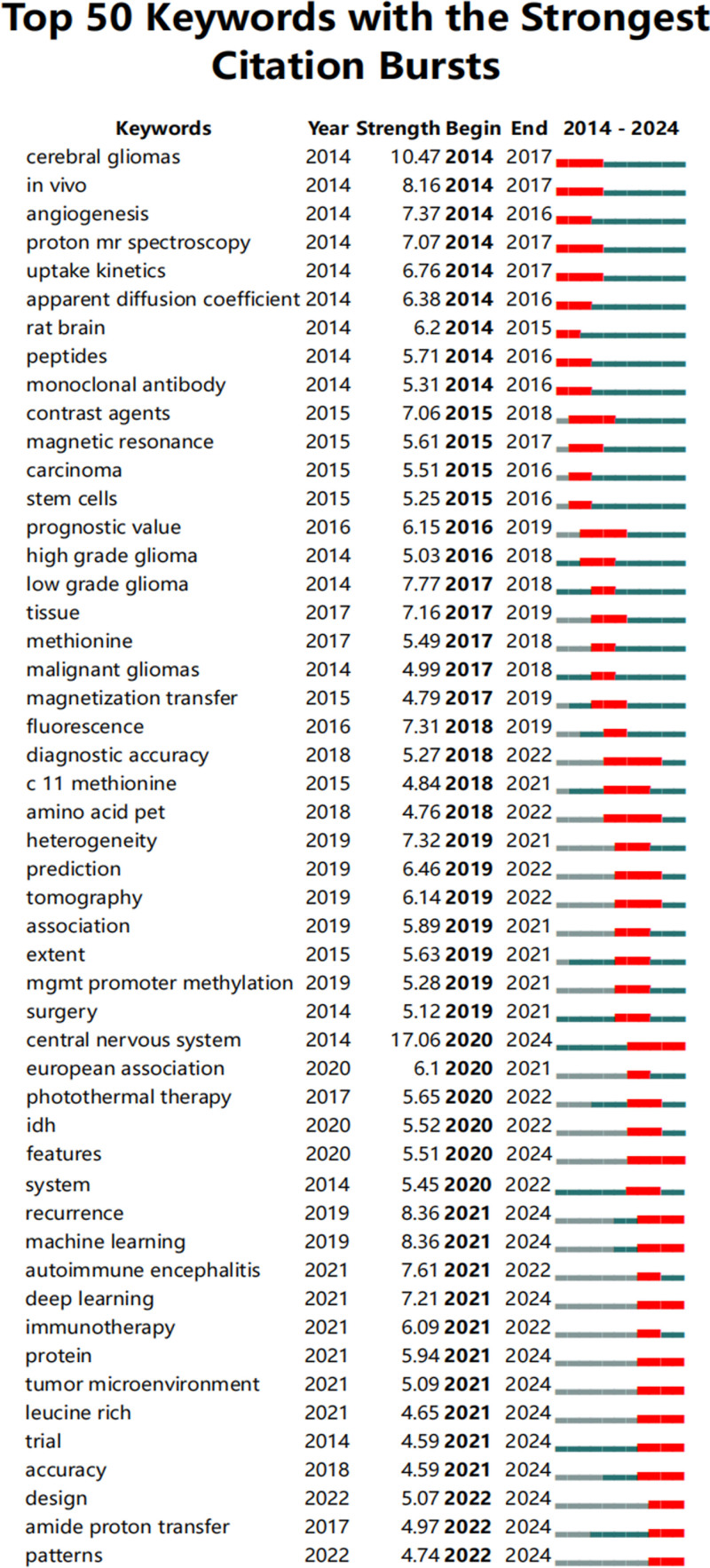
Distribution of journals publishing glioma molecular imaging studies. The bar chart presents the top journals ranked by the number of publications, highlighting the core platforms contributing to the dissemination of research in this field.

**Figure 6.**
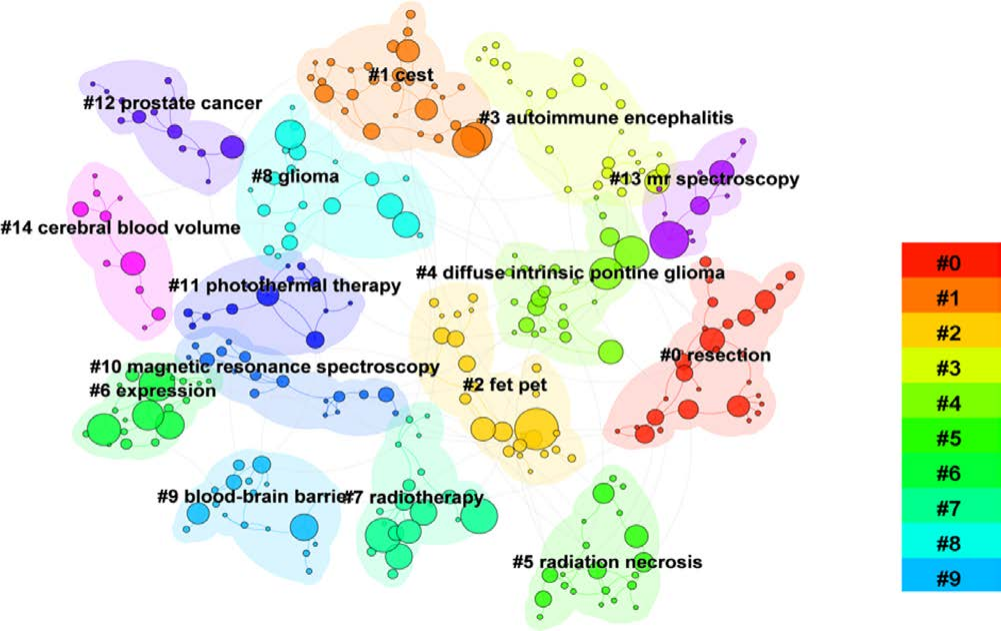
Keyword co-occurrence network in glioma molecular imaging literature. Nodes represent keywords, with larger nodes indicating higher frequency. Links between nodes reflect co-occurrence relationships, and clustering reveals major research themes and topic evolution.

Keyword frequency, burst strength, and clustering analyses indicated the primary focus and evolving trends in glioma molecular imaging (Table 7, Figs. 5 and 6). High-frequency keywords included “PET” and “MRS,” ranked first and second with occurrences of 496 and 391. Notable bridging keywords based on betweenness centrality were “survival” (0.28) and “expression” (0.08).

**Table 7:** Top 20 keywords in glioma molecular imaging publications from 2014 to 2024, ranked by frequency of occurrence.

Rank	Keywords	Occurrences	Centrality	Rank	Keywords	Occurrences	Centrality
1	PET	496	0.02	11	MRI	236	0.11
2	MRS	391	0.16	12	Brain	209	0
3	Brain tumors	342	0.15	13	Glioblastoma multiforme	203	0
4	Glioblastoma	337	0.04	14	Therapy	190	0.06
5	Cancer	326	0.07	15	Radiotherapy	189	0
6	Tumors	317	0.05	16	Classification	184	0.06
7	PET	297	0	17	Central nervous system	158	0.09
8	Survival	282	0.28	18	High grade gliomas	156	0.56
9	In vivo	268	0.07	19	Diagnosis	156	0
10	Expression	259	0.07	20	Gliomas	155	0

Centrality scores reflect the keywords’ importance in the overall co-occurrence network.

MRI = magnetic resonance imaging, MRS = magnetic resonance spectroscopy, PET = positron emission tomography.

Citation burst analysis (Fig. 5) revealed that early research (2014–2017) emphasized foundational topics such as “cerebral gliomas” and “angiogenesis.” From 2018 onward, attention shifted toward practical applications, exemplified by “diagnostic accuracy” and “MGMT promoter methylation.” More recent trends (2022–2024) featured emerging imaging technologies (e.g., “photoacoustic imaging”) and interest in the “tumor microenvironment.”

Keyword clustering (Fig. 6) further delineated specific research themes: Cluster #0 (resection): Surgical interventions and imaging guidance. Cluster #2 (FET PET): Advancements in metabolic imaging for tumor characterization. Cluster #7 (radiotherapy): Imaging’s role in planning and monitoring treatment efficacy. Cluster #11 (photothermal therapy) and Cluster #9 (blood–brain barrier): emerging topics pointing to novel therapeutic and imaging strategies. These clusters illustrate the field’s multidisciplinary nature, integrating imaging modalities, therapeutic strategies, and molecular insights to improve glioma diagnosis and treatment.

## 4. Discussion

Over the past decade (2014–2024), glioma molecular imaging has experienced transformative developments driven by technological innovations and multidisciplinary collaborations.^[20–25]^ These advances have significantly influenced modern medicine, informing clinical decision-making and deepening knowledge of tumor biology.

### 4.1. Temporal trends in research output

Publication trends (Fig. 2) reveal a steady increase from 2014, peaking in 2021. Despite a slight decline in the number of publications, citation counts continued to rise, reaching 11,188 in 2022. This underscores ongoing scholarly interest in glioma molecular imaging and the lasting impact of emerging innovations on diagnostic and therapeutic approaches.

### 4.2. PET imaging: evolution of tracers and applications

PET imaging remains a cornerstone of glioma research, as indicated by its prominence in keyword analyses (Table 7) and co-citation clusters (Fig. 3). While early studies primarily employed FDG tracers, newer amino acid tracers such as F-18-FET and F-18-FDOPA have enhanced glioma by reducing uptake in normal brain tissue.^[26]^ These tracers facilitate more precise differentiation of glioma subtypes^[27–29]^ and underscore the importance of PET-based biomarkers in routine clinical practice. Additionally, PET tracers targeting hypoxia and angiogenesis have expanded PET’s clinical utility, aiding in therapy-response monitoring and clarifying tumor biology.^[30,31]^ Hybrid modalities such as PET/MRI have also gained traction, merging metabolic and structural insights to refine treatment planning.^[32,33]^

## 5. MRI-based innovations

MRS and CEST have substantially enriched MRI-based approaches for glioma assessment.^[34]^ Both techniques offer unique perspectives on tumor metabolism and heterogeneity, as evidenced by their centrality in co-citation analyses (Fig. 3). CEST imaging, for example, enables noninvasive metabolic profiling.^[35]^ Moreover, integrating radiomics and machine learning has improved the accuracy of genomic marker predictions (e.g., IDH mutation, MGMT promoter methylation),^[36–38]^ marking a turning point toward precision medicine.

## 6. Ultrasound: innovations in drug delivery and imaging

Ultrasound technology, particularly focused ultrasound (FUS), addresses longstanding barriers in glioma management by temporarily modulating the blood–brain barrier.^[39]^ This innovation permits targeted delivery of diagnostic probes and therapeutics to the tumor microenvironment, representing a significant breakthrough. Ultrasound-based modalities such as microbubble-enhanced imaging^[40]^ and photoacoustic imaging^[41]^ have further expanded real-time visualization of tumor margins, vascularity, and oxygenation levels, reflecting the growing integration of ultrasound with other modalities.

## 7. Emerging techniques and future directions

Techniques like fluorescence-guided surgery and nanoparticle-based probes are poised for significant growth. Fluorescence imaging with 5-aminolevulinic acid (5-ALA) has improved intraoperative delineation of tumor margins,^[42]^ while theranostic nanoparticles offer combined diagnostic and therapeutic capabilities.^[43]^ These developments underscore the broader trend toward converging diagnostics and therapy, thereby bridging gaps between preoperative planning and intraoperative decision-making.

Bibliometric indicators also highlight a shift toward merging multimodal imaging with omics data. Hybrid systems such as PET/MRI and artificial intelligence (AI)-driven radiomics models offer new avenues to enhance reproducibility, diagnostic accuracy, and clinical applicability.^[44]^ These technologies promise to embed molecular imaging in the broader precision medicine framework firmly.

### 7.1. Summary and implications

Over the past decade, glioma molecular imaging has evolved from traditional imaging methods to highly integrated, multidisciplinary applications. Progress in tracer development, hybrid imaging modalities, and computational analytics has transformed clinical practice, advancing from simple diagnosis to personalized treatment and prognostic management. Challenges persist, including the need for protocol standardization and rigorous validation of AI models. Nonetheless, the synergy of imaging technologies with molecular and genomic data provides unprecedented opportunities to improve clinical outcomes in glioma management, reinforcing the pivotal role of molecular imaging in the future of neuro-oncology.

## 8. Conclusion

In the past decade, molecular imaging has catalyzed significant advancements in glioma research, particularly in noninvasive diagnostics, individualized treatment planning, and therapeutic monitoring. The widespread adoption of hybrid imaging systems, cutting-edge tracers, and AI-driven radiomics has facilitated robust tumor characterization and the prediction of key biomarkers such as IDH mutations and MGMT promoter methylation. While ongoing efforts to standardize protocols and broaden accessibility remain essential, the convergence of imaging technologies and molecular insights signifies a transformative era in neuro-oncology, one that promises to enhance precision medicine and improve patient outcomes.

### 8.1. Limitations

This study has several limitations. First, data collection was limited to the Web of Science Core Collection, which, despite its comprehensive coverage, may not include all pertinent studies from other databases. Second, restricting the search to English-language publications excludes relevant work in different languages, possibly limiting global scope. Finally, although bibliometric methods effectively highlight quantitative trends and collaborations, they do not capture individual studies’ full qualitative depth or clinical impact. Future research could benefit from integrating multiple databases and utilizing a wider range of bibliometric tools to offer a more holistic view of progress in glioma molecular imaging.

## Author contributions

**Methodology:** Hui Zhou.

**Supervision:** Yilin Luo, Shiguang Li.

**Visualization:** Guoping Zhang.

**Writing – original draft:** Hui Zhou.

**Writing – review & editing:** Hui Zhou, Xianchun Zeng.
